# Rehabilitation during a pandemic: psychiatrists as first responders?

**DOI:** 10.1192/bjo.2021.484

**Published:** 2021-06-18

**Authors:** Jeremy Cave, Matthieu Crews

**Affiliations:** 1 Core Trainee, South London and Maudsley NHS Foundation Trust; 2 South London and Maudsley NHS Foundation Trust

## Abstract

**Aims:**

The South London and Maudsley High Support Rehabilitation Team supports a cohort of 120 long-term rehabilitation patients in the densely populated London borough of Southwark.

COVID-19 has a high transmission rate and is more lethal amongst the elderly, ethnic minorities and those with comorbidities.

For these reasons, COVID-19 poses a particular challenge to our patients. Most have significant comorbidities, live communally, engage infrequently with primary care and take high-risk medications like clozapine. Many are from black and minority ethnic backgrounds.

During the Spring coronavirus wave, we found that unwell patients or their carers would contact our service for advice ahead of 111, primary care or emergency services.

In response we designed a standard operating procedure to guide our response to possible cases. This aimed to ensure our advice and management for patients drew upon the latest emerging evidence.

We audited our work and the burden of disease within our service until November 2020.

**Method:**

At a team level, we introduced same-day remote assessments structured around a standard operating procedure incorporating the latest primary care and national guidelines.

At a trust level, treatment guidelines were amended permitting consultant discretion when deciding whether an urgent blood count was required for those unwell on clozapine, and routine blood count monitoring was extended to 3 months for eligible patients

**Result:**

By November 2020 we had only one confirmed case of COVID-19 on our caseload. This patient required ITU and recovered. Seven patients were judged ‘suspected’ to have suffered COVID-19 and eight were possible cases. One supported living accommodation had a possible outbreak.

**Conclusion:**

We are surprised to have had just one confirmed case of COVID-19, despite the vulnerability of our cohort. The attentiveness of our patients and their carers to government guidelines will have contributed to this figure. They have shown remarkable resilience.

This pandemic has prompted trust-wide changes to clozapine monitoring and perhaps a permanently less intensive monitoring regime for some patients.

That our patients contacted our team ahead of 111, primary care or emergency services may reflect the close trust they place in us to support them through difficulty. It is fitting for a service aiming to provide holistic care that our scope should have expanded in this way during the pandemic. Community rehabilitation services are well placed to act as first responders.

